# GFAT1/HBP/O-GlcNAcylation Axis Regulates *β*-Catenin Activity to Promote Pancreatic Cancer Aggressiveness

**DOI:** 10.1155/2020/1921609

**Published:** 2020-02-15

**Authors:** Chunzeng Jia, Hengchao Li, Deliang Fu, Yu Lan

**Affiliations:** ^1^Department of Gastroenterology, Beijing Jishuitan Hospital, Beijing 100035, China; ^2^Department of Pancreatic Surgery, Huashan Hospital, Fudan University, Shanghai 200040, China

## Abstract

Reprogrammed glucose and glutamine metabolism are essential for tumor initiation and development. As a branch of glucose and metabolism, the hexosamine biosynthesis pathway (HBP) generates uridine diphosphate N-acetylglucosamine (UDP-GlcNAc) and contributes to the O-GlcNAcylation process. However, the spectrum of HBP-dependent tumors and the mechanisms by which the HBP promotes tumor aggressiveness remain areas of active investigation. In this study, we analyzed the activity of the HBP and its prognostic value across 33 types of human cancers. Increased HBP activity was observed in pancreatic ductal adenocarcinoma (PDAC), and higher HBP activity predicted a poor prognosis in PDAC patients. Genetic silencing or pharmacological inhibition of the first and rate-limiting enzyme of the HBP, glutamine:fructose-6-phosphate amidotransferase 1 (GFAT1), inhibited PDAC cell proliferation, invasive capacity, and triggered cell apoptosis. Notably, these effects can be restored by addition of UDP-GlcNAc. Moreover, similar antitumor effects were noticed by pharmacological inhibition of GFAT1 with 6-diazo-5-oxo-l-norleucine (DON) or Azaserine. PDAC is maintained by oncogenic Wnt/*β*-catenin transcriptional activity. Our data showed that GFAT1 can regulate *β*-catenin expression via modulation of the O-GlcNAcylation process. TOP/FOP-Flash and real-time qPCR analysis showed that GFAT1 knockdown inhibited *β*-catenin activity and the transcription of its downstream target genes *CCND1* and *MYC*. Ectopic expression of a stabilized form of *β*-catenin restored the suppressive roles of GFAT1 knockdown on PDAC cell proliferation and invasion. Collectively, our findings indicate that higher GFAT1/HBP/O-GlcNAcylation exhibits tumor-promoting roles by maintaining *β*-catenin activity in PDAC.

## 1. Introduction

Reprogrammed energy metabolism is emerged as a hallmark of cancer cells [1]. Different from normal cells, cancer cells preferentially utilize glycolysis instead of oxidative phosphorylation to produce energy even in the presence of sufficient oxygen, a phenomenon called aerobic glycolysis, also known as the Warburg effect [2, 3]. In addition, cancer cells are addicted to the amino acid glutamine to fuel anabolic processes [4]. As a branch of glucose and glutamine metabolism, the hexosamine biosynthesis pathway (HBP) consumes approximately 2-5% of the total glucose and a small fraction of glutamine to generate uridine diphosphate N-acetylglucosamine (UDP-GlcNAc) [5]. UDP-GlcNAc is profoundly implicated in the classical glycosylation and O-GlcNAcylation process, which posttranslationally modifies many cytosolic and nuclear proteins by O-linked-N-acetylglucosamine (O-GlcNAc) [6]. Although O-GlcNAcylation has been reported to link the HBP to malignant activities, its activity and prognostic value in human cancers remain largely unexplored [7–9].

The first and rate-limiting step of the HBP is catalyzed by glutamine:fructose-6-phosphate amidotransferase (GFAT) that converts fructose-6-phosphate to glucosamine-6-phosphate [10, 11]. There are two different GFAT genes, GFAT1 and GFAT2, and GFAT1 is the major form ubiquitously expressed in human tissues. Previously, aberrant expression of GFAT1 has been demonstrated in several cancers, including hepatocellular carcinoma and pancreatic ductal adenocarcinoma (PDAC) [12, 13]. High GFAT1 expression is identified as an independent predictor of adverse clinical outcome for PDAC patients [12]. Moreover, many factors have been reported to regulate GFAT1 expression and activity in cancers, such as mTOR complex, AMPK, and c-Myc [11, 14, 15]. However, the cellular oncogenic roles of GFAT1 and its underlying mechanism in PDAC are not clear.

In this study, we performed a pan-cancer analysis of the HBP and uncovered that HBP activity is upregulated in PDAC and increased HBP activity predicts a poor prognosis in PDAC patients. Blockade of HBP activity by genetic or pharmacological inhibition of GFAT1 significantly suppressed PDAC cell proliferation and invasive potential. Mechanistically, we revealed that *β*-catenin activity was maintained by GAFT1-mediated O-GlcNAcylation. The activity of Wnt/*β*-catenin signaling and expression of its downstream targets were suppressed by GFAT1 knockdown.

## 2. Materials and Methods

### 2.1. Cell Culture and Reagent

The pancreatic cancer cell lines AsPC1, BxPC3, Capan1, MiaPaca2, PANC1, and SW1990 used in this study were all obtained from the Institute of Biochemistry and Cell Biology, Chinese Academy of Science (Shanghai, China). All cells were cultured in Dulbecco's modified Eagle's medium (DMEM, Gibco) or Roswell Park Memorial Institute- (RPMI-) 1640 medium supplemented with 10% fetal bovine serum (*v*/*v*, Gibco, USA) and 1% streptomycin-penicillin (Sigma-Aldrich, Shanghai, China). All cells were incubated in a humidified atmosphere with 5% CO_2_ at 37°C. LY294002, rapamycin, and ICG-001 were obtained from Selleck (Shanghai, China). The GFAT1 inhibitor 6-diazo-5-oxo-L-norleucine (DON) and Azaserine were purchased from Sigma-Aldrich (Shanghai, China). OSMI-1 was purchased from Selleck (SM1621, Sigma-Aldrich, Shanghai, China). OSMI-1 was treated at 30 *μ*M for 24 h before functional experiment analysis.

### 2.2. Online Data Analysis

For pan-cancer analysis of the activity of the hexosamine biosynthetic pathway (HBP) and its prognostic value, data derived from The Cancer Genome Atlas (TCGA, http://www.tcga.org/) database was used. The online available analyzer GEPIA2 (http://gepia2.cancer-pku.cn/) was conducted for correlation analysis. The mean value of the log2(TPM + 1) is used to calculate the signature score as reported previously [16].

### 2.3. RNA Interference and Gene Overexpression

Two specific siRNAs against Glutamine-Fructose-6-Phosphate Transaminase 1 (GFAT1) and the *β*-catenin S33Y plasmid were designed and synthesized in GenePharma Co., LTD. (Shanghai, China). The negative control was nonhomologous to any human genome sequences. In brief, BxPC-3 and SW1990 cells were seeded in 6-well plates at a density of 3‐5 × 10^5^ cells per well. When reached 50-70% confluence, cells were transfected with siRNAs or overexpression vector using RNAimax or Lipofectamine (Thermo Fisher Scientific, USA) according to the manufacturer's instructions. After incubation for 48 h, cells were collected to analyze the knockdown efficiency or subjected for indicated cell experiments.

### 2.4. Western Blotting Analysis

Cell total protein was extracted using RIPA lysis buffer containing protease inhibitor cocktail (Beyotime, China). The protein concentration was detected using a BCA Protein Assay Kit (Pierce Biotechnology). Thirty micrograms of protein were separated using 6-10% SDS-PAGE (*v*/*v*) and transferred to nitrocellulose membranes (Millipore, MA, USA). Then, the nitrocellulose membranes were blocked with 5% nonfat milk, followed by probing corresponding primary antibodies: anti-mTOR (#2983, 1 : 1,000; Cell Signaling Technology), anti-p-mTOR (#2971, 1 : 1,000; Cell Signaling Technology), anti-Akt (#4685, 1 : 1000; Cell Signaling Technology), anti-p-Akt (#4060, 1 : 2,000; Cell Signaling Technology), anti-*β*-catenin (ab32572, 1 : 2000; Abcam), anti-O-GlcNAc (ab2739, 1 : 1000; Abcam), and anti-*β*-actin antibody (ab8226, 1 : 2000; Abcam). After incubation with primary antibodies overnight at 4°C, the membranes were incubated the horseradish peroxidase-conjugated secondary antibodies in the next day. Finally, the antigen-antibody complexes were detected by enhanced chemiluminescence (Pierce, USA). Protein quantification was performed by ImageJ software (NIH Image), and the *β*-actin was used as an internal control.

### 2.5. Immunoprecipitations

Cell total protein was immunoprecipitated from whole-cell extracts. Briefly, cells were collected, washed, and lysed on ice for 15 min in an IP lysis buffer containing protease inhibitor cocktail (Beyotime, China). Protein concentration from cell supernatants was estimated by a BCA Protein Assay Kit (Pierce Biotechnology). Whole-cell extracts were subjected to immunoprecipitation with protein A/G-linked *β*-catenin antibody or control IgG, followed by the immunoblotting with indicated proteins. Then, eluted proteins were analyzed by Western blotting as described above.

### 2.6. Cell Proliferation and Apoptosis Assay

The Cell Counting Kit (CCK8, Dojindo, Japan) was used to determine cell viability. In brief, cells were seeded into 96-well plates in triplicate at a density of 3,000 cells per well. Cell viability was measured every 24 h using the CCK-8 according to the manufacturer's instructions. The absorbance at 450 nm was measured using a multifunctional microplate reader (Bio-Rad). For cell apoptosis, indicated cells were starved for 48 h and then cells were collected and subjected for Apo-ONE Homogeneous Caspase-3/7 Assay (Promega, USA). The presented data were normalized to protein content.

### 2.7. Cell Invasion Assay

Transwell invasion assays were performed in 24-well transwell plates (8 *μ*m) according to the manufacturer's instructions (Corning, New York, NY, USA). Before experiment, 100 *μ*l Matrigel (BD Biosciences, USA) was preincubated in the upper chamber and allowed to solidification. Then, the upper chamber was incubated with 1 × 10^4^ cells in 100 *μ*l culture medium without serum. The lower chamber was supplemented with 700 *μ*l culture medium containing 10% FBS (*v*/*v*). After incubation for 24 h at 37°C, the noninvaded cells were removed, and the invaded cells on the lower surface were fixed with 4% paraformaldehyde (*w*/*v*) and stained with 0.25% crystal violet (*w*/*v*). The number of invaded cells was counted in six randomly selected microscopic fields.

### 2.8. Real-Time Quantitative PCR

The methods for real-time qPCR analysis is reported elsewhere [17]. In brief, total RNA was isolated from PDAC cells by using the Trizol reagent (Invitrogen, USA) and reverse transcribed were performed using the PrimeScript RT-PCR Kit (Takara, Japan). The mRNA levels were quantified on an ABIPrism-7500 Sequence Detector System (ABI, Applied Biosystems, USA). The ACTB was used as an internal control. Relative mRNA expression of indicated genes was normalized to the expression of ACTB (*β*-actin). All reactions were run in triplicate. The primer sequences were shown as follows: *GFAT1* forward, 5′-GGAATAGCTCATACCCGTTGG-3′; *GFAT1* reverse, 5′-TCGAAGTCATAGCCTTTGCTTT-3′; *CCND1* forward, 5′-CAATGACCCCGCACGATTTC-3′; *CCND1* reverse, 5′-CATGGAGGGCGGATTGGAA-3′; *MYC* forward, 5′-GTCAAGAGGCGAACACACAAC-3′; *MYC* reverse, 5′-TTGGACGGACAGGATGTATGC-3′; *ACTB* forward, 5′-CATGTACGTTGCTATCCAGGC-3′; and *ACTB* reverse, 5′-CTCCTTAATGTCACGCACGAT-3′.

### 2.9. Luciferase Reporter Assay

Briefly, BxPC-3 and SW1990 cells were transfected with si-ctrl and si-GFAT1 in combination with the TOP-Flash and FOP-Flash vectors using Lipofectamine 2000 reagent (Invitrogen, USA) for 24 h. After 24 h incubation, luciferase activity was assessed with the Dual-Luciferase Reporter Assay Kit (Promega, USA). The intensity values of blank wells were substrated from experimental data, and the presented data were normalized to control wells (with control siRNA).

### 2.10. Statistical Analysis

All the experiments in this study were performed in triplicate and repeated at least 3 times. All data are presented as the means ± SDs. Statistical significance was determined by the two-tailed Student's *t*-test or one-way ANOVA test. Statistical analyses were performed by using SPSS 13.0 statistical package software (SPSS, Chicago, IL, USA) or GraphPad Prism (GraphPad Software Inc., San Diego, CA). *P* values of less than 0.05 were considered statistically significant.

## 3. Results

### 3.1. Increased HBP Activity Predicts a Poor Prognosis in PDAC

**Figure 1:**
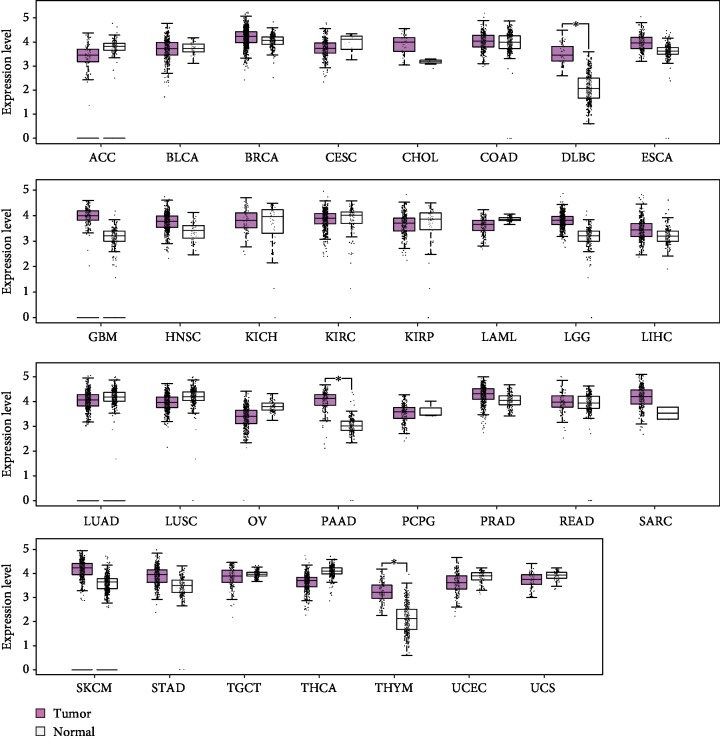
Pan-cancer analysis of HBP activity in 31 tumor types. The dataset sources and cancer annotations are available at http://gepia2.cancer-pku.cn/. ^∗^*P* < 0.05. ACC: adrenocortical carcinoma; BLCA: Bladder Urothelial Carcinoma; BRCA: breast invasive carcinoma; CESC: cervical squamous cell carcinoma and endocervical adenocarcinoma; CHOL: cholangiocarcinoma; COAD: colon adenocarcinoma; DLBC: Lymphoid Neoplasm Diffuse Large B-cell Lymphoma; ESCA: esophageal carcinoma; GBM: glioblastoma multiforme; HNSC: head and neck squamous cell carcinoma; KICH: kidney chromophobe; KIRC: kidney renal clear cell carcinoma; KIRP: kidney renal papillary cell carcinoma; LAML: Acute Myeloid Leukemia; LGG: Brain Lower Grade Glioma; LIHC: liver hepatocellular carcinoma; LUAD: lung adenocarcinoma; LUSC: lung squamous cell carcinoma; OV: ovarian serous cystadenocarcinoma; PAAD: pancreatic adenocarcinoma; PCPG: Pheochromocytoma and Paraganglioma; PRAD: prostate adenocarcinoma; READ: rectum adenocarcinoma; SARC: sarcoma; SKCM: Skin Cutaneous Melanoma; STAD: stomach adenocarcinoma; TGCT: Testicular Germ Cell Tumors; THCA: thyroid carcinoma; THYM: thymoma; UCEC: Uterine Corpus Endometrial Carcinoma; UCS: Uterine Carcinosarcoma.

A 14-gene signature (*B4GALNT2*, *DPAGT1*, *SLC35A3*, *GFAT1*, *MGAT1*, *AMDHD2*, *PGM3*, *NAGK*, *RENBP*, *GNPNAT1*, *GNPDA1*, *UAP1*, *UAP1L1*, and *GFAT2*) from the Molecular Signatures Database (MSigDB, http://software.broadinstitute.org/gsea/msigdb/) was used for determining HBP activity. Combined the data from The Cancer Genome Atlas (TCGA) and Genotype-Tissue Expression (GTEx), we calculated a HBP score for each sample and found that HBP activity was significantly and specifically increased in diffuse large B-cell lymphoma (DLBC), pancreatic adenocarcinoma (PAAD), and thymoma (THYM) compared with their normal counterparts (Figure 1). Using the median HBP score as a cutoff, we investigated the prognostic value of HBP activity in 33 tumor types. As a result, increased HBP activity was closely associated with an adverse clinical outcome in adrenocortical carcinoma (ACC), breast invasive carcinoma (BRCA), kidney chromophobe (KICH), lower grade glioma (LGG), liver hepatocellular carcinoma (LIHC), lung adenocarcinoma (LUAD), and PAAD (Table 1). Notably, elevated HBP activity predicted a better prognosis in kidney renal clear cell carcinoma (KIRC) (Table 1). Of note, HBP activity was increased in PDAC and predicted a poor prognosis in PDAC patients. Therefore, we focused on the study of HBP in PDAC.

### 3.2. Increased HBP Activity Is Associated with PDAC Malignancies

**Figure 2:**
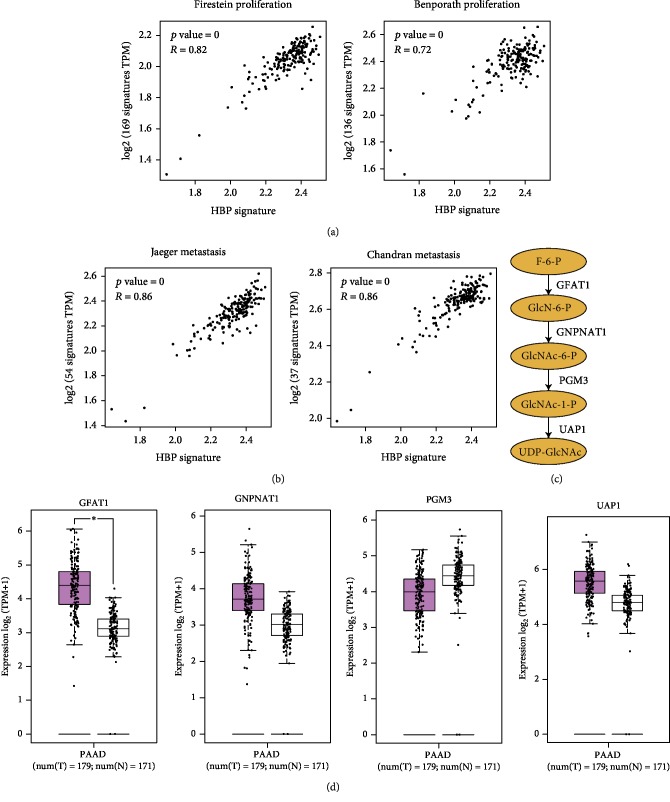
Increased HBP activity is associated with PDAC malignancies. (a, b) Correlation analysis of HBP gene signatures and tumor cell proliferation and metastasis gene signatures. (c) Overview of the HBP. (d) Expression pattern of key HBP components in PDAC. ^∗^*P* < 0.05.

By correlation analysis, we found that the HBP gene signature was closely associated with the tumor cell proliferation (Figure 2(a)) and metastasis (Figure 2(b)). GFAT1 is the first and rate-limiting enzyme of HBP; glucosamine-phosphate N-acetyltransferase 1 (GNPNAT1) catalyzes the acetylation step of HBP [7]; UDP-N acetylglucosamine pyrophosphorylase 1 (UAP1) catalyzes the final enzymatic reaction of HBP [18], contributing to UDP-GlcNAc synthesis (Figure 2(c)). In PDAC, we revealed a slight increase of GNPNAT1 and UAP1 expression and a significant overexpression of GFAT1 in tumor tissues compared with normal counterparts (Figure 2(d)). Thus, we aimed to determine the potential oncogenic roles of GFAT1 in PDAC.

### 3.3. GFAT1 Knockdown or Inhibition Suppresses PDAC Aggressiveness

**Figure 3:**
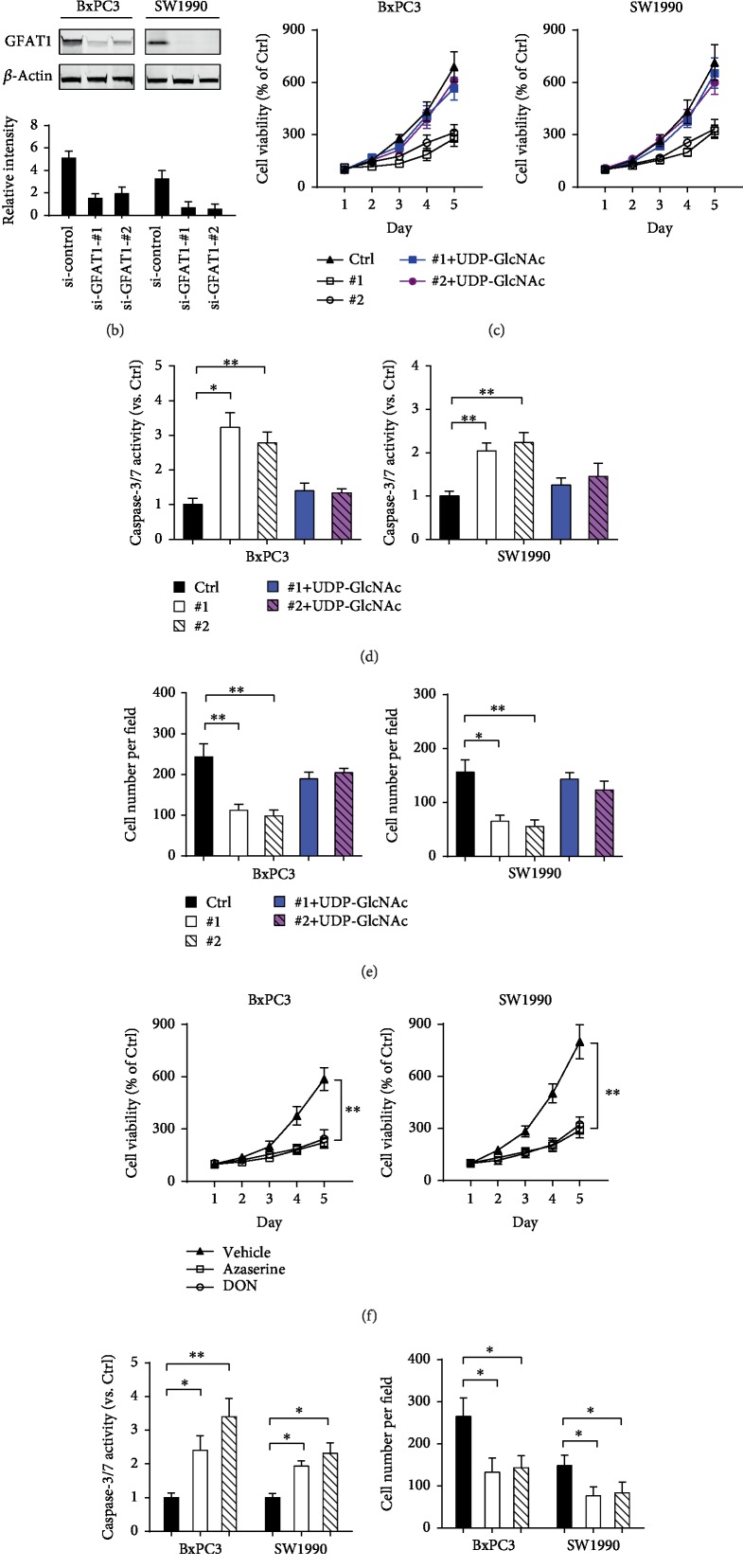
GFAT1 knockdown or inhibition suppresses PDAC aggressiveness. (a) Western blotting analysis of the GFAT1 protein level in PDAC cell lines; quantification data was shown, and the *β*-actin was used as an internal control. (b) Western blotting analysis of the knockdown efficiency of GFAT1 in BxPC3 and SW1990 cells; quantification data was shown, and the *β*-actin was used as an internal control. (c) The effect of GFAT1 knockdown on BxPC3 and SW1990 cell proliferation was determined by CCK-8 assay. (d) The effect of GFAT1 knockdown on BxPC3 and SW1990 cell apoptosis was determined by caspase-3/7 activity assay. (e) The effect of GFAT1 knockdown on BxPC3 and SW1990 cell invasion was determined by transwell assay. (f) The effect of GFAT1 inhibition on BxPC3 and SW1990 cell proliferation was determined by CCK-8 assay. (g) The effects of GFAT1 inhibition on BxPC3 and SW1990 cell apoptosis and invasion were determined by caspase-3/7 activity assay and transwell assay, respectively. ^∗^*P* < 0.05; ^∗∗^*P* < 0.01.

To test if increased HBP activity contributes to PDAC aggressiveness, loss-of-function study in GFAT1 was performed. Two cell lines with higher endogenous level of GFAT1, BxPC3 and SW1990, were subjected for knockdown experiments (Figure 3(a)). Two specific siRNAs against GFAT1 led to remarkable reduction in the GFAT1 protein level (Figure 3(b)). By cell viability assay, we showed that GFAT1 knockdown resulted in decreased PDAC cell proliferation (Figure 3(c)). At the same time, GFAT1 knockdown also augmented starvation-induced cell apoptosis in PDAC as revealed by increased caspase-3/7 activity (Figure 3(d)). In addition, the invasive potential of PDAC cells was largely compromised by GFAT1 knockdown (Figure 3(e)). Interestingly, addition of 50 mM UDP-GlcNAc was sufficient to restore the inhibitory effect on cell proliferation and invasive capacity (Figures 3(c)–3(e)). Moreover, pharmacological inhibition of GFAT1 with 6-diazo-5-oxo-l-norleucine (DON, 50 *μ*M) or Azaserine (50 *μ*M) also significantly inhibited cell proliferation and invasive potential and triggered cell apoptosis (Figures 3(f) and 3(g)). Collectively, these data above suggest that GFAT1 blockade can impair PDAC cell proliferation and invasion as well as promote cell apoptosis by downregulating HBP activity.

### 3.4. GFAT1-Mediated O-GlcNAcylation Promotes *β*-Catenin Activity

**Figure 4:**
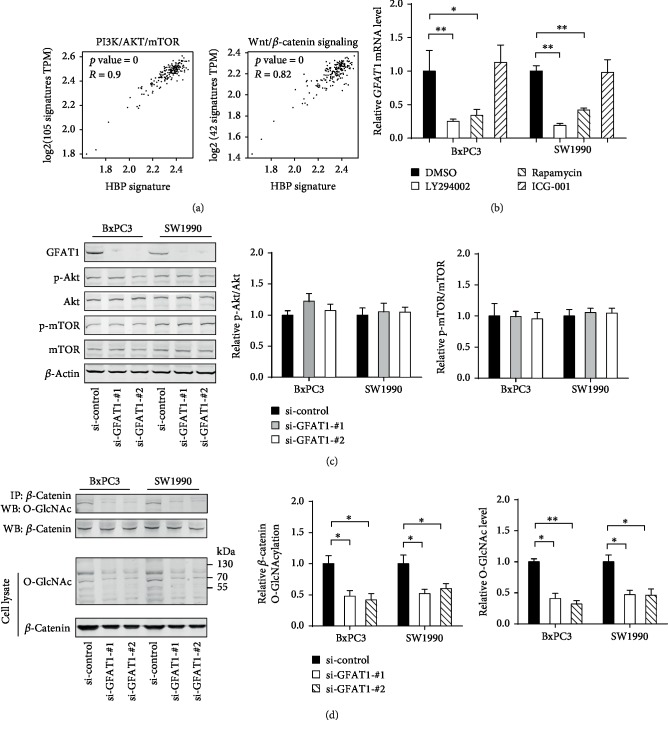
GFAT1 is induced by the PI3K/AKT/mTOR pathway and promotes *β*-catenin activity by O-GlcNAcylation. (a) Correlation analysis of HBP gene signatures and PI3K/AKT/mTOR and Wnt/*β*-catenin signaling gene signatures. (b) The effects of LY294002 (20 *μ*M), rapamycin (50 nM), and ICG-001 (5 *μ*M) on the mRNA expression of GFAT1 in BxPC3 and SW1990 cells were analyzed by real-time qPCR. (c) Western blotting analysis of the effect of GFAT1 knockdown on the PI3K/AKT/mTOR signaling in BxPC3 and SW1990 cells; quantification data was shown, and the *β*-actin was used as an internal control. (d) Western blotting analysis of the effect of GFAT1 knockdown on the *β*-catenin O-GlcNAcylation; quantification data was shown, and the *β*-actin was used as an internal control. ^∗^*P* < 0.05; ^∗∗^*P* < 0.01.

**Figure 5:**
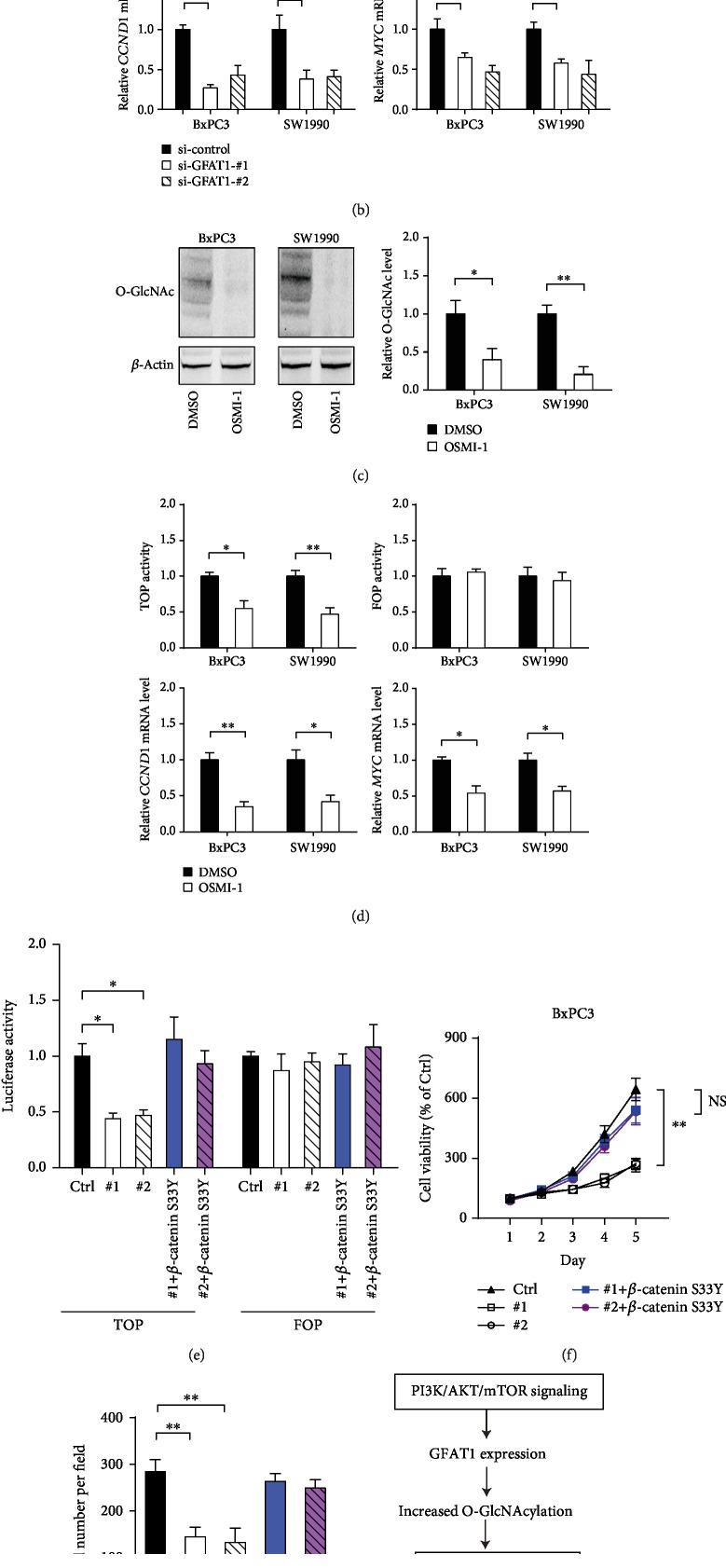
*β*-Catenin mediates the oncogenic roles of GFAT1 in PDAC cells. (a) The effect of GFAT1 knockdown on the *β*-catenin activity in BxPC3 and SW1990 cells was detected by luciferase TOP/FOP activity experiment. (b) The effect of GFAT1 knockdown on the cyclin D1 and c-Myc mRNA expression was measured by real-time qPCR. (c) Western blotting analysis of the level of O-GlcNAcylation upon OSMI-1 treatment; quantification data was shown, and the *β*-actin was used as an internal control. (d) The effect of OSMI-1 treatment on *β*-catenin activity and its downstream targets in BxPC3 and SW1990 cells. (e–g) The effect of GFAT1 knockdown on BxPC3 cell proliferation and invasion upon introduction of *β*-catenin S33Y mutant plasmid. (h) Activated PI3K/AKT/mTOR signaling promotes GFAT1 expression, which increases the production of UDP-GlcNAc, inducing aberrant stability of *β*-catenin. Finally, *β*-catenin promotes tumor growth and metastasis through downstream targets, such as CCND1 and c-Myc. ^∗^*P* < 0.05; ^∗∗^*P* < 0.01.

Using the Hallmark gene sets from MSigDB, we found that HBP gene signature was positively associated with the activity of PI3K/AKT/mTOR and Wnt/*β*-catenin signaling (Figure 4(a)). This result indicated that increased HBP activity might be mediated by PI3K/AKT/mTOR and Wnt/*β*-catenin signaling or PDAC cell-specific O-GlcNAcylation may impinge on oncogenic PI3K/AKT/mTOR and Wnt/*β*-catenin signaling. To uncover this issue, we blocked PI3K/AKT/mTOR and Wnt/*β*-catenin signaling with specific small molecular inhibitors. As a result, LY294001 and rapamycin dramatically inhibited *GFAT1* mRNA expression, while the inhibitor of Wnt/*β*-catenin signaling ICG-001 showed no significant influence, suggesting that PI3K/AKT/mTOR signaling may contribute to HBP activity by inhibition of GFAT1 expression in PDAC (Figure 4(b)). Next, we found that GFAT1 knockdown did not affect PI3K/AKT/mTOR signaling activity as revealed by the phosphorylation level of AKT and mTOR (Figure 4(c)). Surprisingly, we noticed that GFAT1 knockdown in PDAC cells reduced *β*-catenin O-GlcNAcylation (Figure 4(d)). To further confirm this observation, we used the TOP/FOP FLASH assay to evaluate *β*-catenin activity upon genetic silencing of GFAT1. As shown in Figure 5(a), GFAT1 knockdown led to a half reduction in TOP/FOP activity in BxPC3 and SW1990. Moreover, the expression of known downstream targets of Wnt/*β*-catenin signaling, *CCND1* and *MYC*, was significantly downregulated by GFAT1 knockdown (Figure 5(b)). UDPGlcNAc is not only the substrate for protein OGlcNAcylation but also the substrate for N-glycosylation and glycosaminoglycan biosynthesis. Therefore, we further tested the effect of O-linked-*β*-N-acetylglucosamine transferase (OGT) inhibition by OSMI-1 (Figure 5(c)). As a result, OSMI-1 significantly reduced the TOP/FOP activity and the expression of *β*-catenin downstream targets (Figure 5(d)). Moreover, ectopic expression, a stabilized form of *β*-catenin (*β*-catenin Ser33Y), largely rescued the inhibitory effect of GFAT1 knockdown on cell proliferation and invasion (Figures 5(e)–5(g)) of BxPC3 cells. Taken together, these findings indicate that PI3K/AKT/mTOR signaling can upregulate GFAT1 expression to promote HBP, which further facilitates Wnt/*β*-catenin signaling activity through enhancing *β*-catenin O-GlcNAcylation to promote PDAC cell proliferation and invasion (Figure 5(h)).

## 4. Discussion

PDAC is a leading cause of cancer-related deaths worldwide with a 5-year survival less than 8% [19]. Like most human cancers, the deaths caused by PDAC are correlated with its clinical stages. However, most PDAC patients are diagnosed at an advanced stage and 80% of PDAC tumors are unresectable [20, 21]. Thus, a better understanding of the drive factors involved in PDAC progression is essential for the development of new therapeutic drugs.

It has been well documented that HBP/O-GlcNAcylation process is critical to tumorigenesis [22]. Accumulated evidence showed that O-GlcNAcylation is involved in many cellular processes, such as epigenetics, mRNA transcription, protein translation, and signaling transduction [23]. In this study, our pan-cancer analysis showed that HBP activity is dysregulated in many cancer types and correlates patients' prognosis, especially in PDAC. As the rate-limiting step of the HBP, GFAT1 expression is closely associated with glycosylation alteration in cancer cells [24]. Of note, GFAT1 is highly expressed in PDAC and predicts a poor prognosis [12]. In PDAC, the predominant KRAS mutation serves a vital role in controlling glucose metabolism through enhancing glucose uptake and channeling of glucose intermediates into the HBP and pentose phosphate pathways (PPP) [25]. KRAS inactivation results in decreased GFAT1 expression and inhibition of the HBP and protein O-glycosylation. Meanwhile, knockdown of GFAT1 leads to reduced O-GlcNAcylation to a similar level induced by KRAS inactivation, suggesting the important roles of GFAT1 in the HBP flux [25]. Hence, targeting GFAT1 might exhibit an antitumor effect by reducing HBP activity. Consistent with notion, GFAT1 deficiency impairs the malignant features of glioblastoma (GBM) and reduces the glucosamine-P-6 synthesis [14]. In cholangiocarcinoma, treatment with the GFAT inhibitor DON reduces global O-GlcNAcylated proteins and inhibits cell migration [26]. In this study, we found that GFAT1 knockdown suppresses tumor cell proliferation and invasion and triggers cell apoptosis, and these effects can be compromised by addition of UDP-GlcNAc. Indeed, reduced HBP flux leads to disordered glycosylation of proteins and subsequently induction of cell death [25]. Meanwhile, the decrease of protein glycosylation is lethal for many cancer cells which need protein glycosylation for oncogenic cellular functions [27].

It has been reported that the PI3K/Akt pathway stimulates glucose metabolism and provides more UDP-GlcNAc [27, 28]. Consistently, we uncovered that increased GFAT1 expression is mediated by the PI3K/Akt/mTOR pathway in PDAC. Previously, many reports suggest that the HBP is involved in the regulation of *β*-catenin [29–33]. For example, increased glucose levels are correlated with endometrial cancer through upregulation of the HBP and O-GlcNAcylation, which enhances the stability of *β*-catenin [31]. Many reports have also revealed that the increased O-GlcNAcylation level is able to increase the *β*-catenin activity, nuclear accumulation, and subsequent transcription of its target genes [31, 34–36]. In colorectal cancer, upregulation of the HBP reverberates on high O-GlcNAcylation levels including modification of *β*-catenin to promote cell proliferation [37]. Here, we found that GFAT1 knockdown-mediated O-GlcNAc reduction inhibits *β*-catenin suggesting that elevated O-GlcNAcylation contributing to PDAC malignant phenotypes likely acts in part through maintenance of the oncogenic Wnt/*β*-catenin signaling pathway.

In conclusion, the present study demonstrated that HBP activity is increased in many human cancers and can act as a prognostic factor. Blockade of GFAT1 function exhibits remarkable antitumor effects via disruption of the HBP flux and *β*-catenin O-GlcNAcylation in PDAC cells. Thus, PDAC cells are addicted to increased HBP activity and suppression of the HBP by targeting GFAT1 may serve as a novel therapeutic intervention in PDAC.

## Figures and Tables

**Table 1:** Pan-cancer analysis of the prognostic value of the hexosamine biosynthetic pathway.

Tumor	High (*n*)	Low (*n*)	HR	*P* value	Tumor	High (*n*)	Low (*n*)	HR	*P* value
ACC	38	38	2.4	0.023	LUSC	241	241	1.0	0.86
BLCA	201	201	1.3	0.13	MESO	41	41	0.95	0.83
BRCA	535	535	1.5	0.007	OV	212	212	1.2	0.17
CESC	146	146	1.3	0.25	PAAD	89	89	1.6	0.02
CHOL	18	18	0.72	0.5	PCPG	91	91	2.4	0.32
COAD	135	135	1.0	0.87	PRAD	246	246	0.77	0.69
DLBC	23	23	1.0	1.0	READ	46	46	1.1	0.87
ESCA	91	91	1.1	0.74	SARC	131	131	1.0	0.92
GBM	81	81	1.3	0.12	SKCM	229	229	1.0	0.84
HNSC	259	259	0.99	0.97	STAD	192	192	1.1	0.7
KICH	32	32	8.5	0.015	TGCT	68	68	1.9	0.61
KIRC	258	258	0.67	0.011	THCA	255	255	1.5	0.41
KIRP	141	141	0.99	0.97	THYM	59	59	1.8	0.26
LAML	53	53	1.1	0.82	UCEC	86	86	1.0	0.92
LGG	257	257	1.8	0.002	UCS	28	28	0.81	0.53
LIHC	182	182	1.6	0.012	UVM	39	39	2.1	0.092
LUAD	239	239	1.4	0.022					

High and low indicate tumor samples with higher and lower HBP activity, respectively. HR is hazard ratio; the *P* value was a comparison of survival time between tumor samples with higher and lower HBP activity by the log-rank test. ACC: adrenocortical carcinoma; BLCA: Bladder Urothelial Carcinoma; BRCA: breast invasive carcinoma; CESC: cervical squamous cell carcinoma and endocervical adenocarcinoma; CHOL: cholangiocarcinoma; COAD: colon adenocarcinoma; DLBC: Lymphoid Neoplasm Diffuse Large B-cell Lymphoma; ESCA: esophageal carcinoma; GBM: glioblastoma multiforme; HNSC: head and neck squamous cell carcinoma; KICH: kidney chromophobe; KIRC: kidney renal clear cell carcinoma; KIRP: kidney renal papillary cell carcinoma; LAML: Acute Myeloid Leukemia; LGG: Brain Lower Grade Glioma; LIHC: liver hepatocellular carcinoma; LUAD: lung adenocarcinoma; LUSC: lung squamous cell carcinoma; MESO: mesothelioma; OV: ovarian serous cystadenocarcinoma; PAAD: pancreatic adenocarcinoma; PCPG: Pheochromocytoma and Paraganglioma; PRAD: prostate adenocarcinoma; READ: rectum adenocarcinoma; SARC: sarcoma; SKCM: Skin Cutaneous Melanoma; STAD: stomach adenocarcinoma; TGCT: Testicular Germ Cell Tumors; THCA: thyroid carcinoma; THYM: thymoma; UCEC: Uterine Corpus Endometrial Carcinoma; UCS: Uterine Carcinosarcoma; UVM: Uveal Melanoma.

## Data Availability

The data used to support the findings of this study are available from the corresponding author upon request.
